# The Distribution of GYR- and YLP-Like Motifs in *Drosophila* Suggests a General Role in Cuticle Assembly and Other Protein-Protein Interactions

**DOI:** 10.1371/journal.pone.0012536

**Published:** 2010-09-02

**Authors:** R. Scott Cornman

**Affiliations:** Department of Cellular Biology, University of Georgia, Athens, Georgia, United States of America; Freie Universitaet Berlin, Germany

## Abstract

**Background:**

Arthropod cuticle is composed predominantly of a self-assembling matrix of chitin and protein. Genes encoding structural cuticular proteins are remarkably abundant in arthropod genomes, yet there has been no systematic survey of conserved motifs across cuticular protein families.

**Methodology/Principal Findings:**

Two short sequence motifs with conserved tyrosines were identified in *Drosophila* cuticular proteins that were similar to the GYR and YLP Interpro domains. These motifs were found in members of the CPR, Tweedle, CPF/CPFL, and (in *Anopheles gambiae*) CPLCG cuticular protein families, and the Dusky/Miniature family of cuticle-associated proteins. Tweedle proteins have a characteristic motif architecture that is shared with the *Drosophila* protein GCR1 and its orthologs in other species, suggesting that GCR1 is also cuticular. A resilin repeat, which has been shown to confer elasticity, matched one of the motifs; a number of other *Drosophila* proteins of unknown function exhibit a motif architecture similar to that of resilin. The motifs were also present in some proteins of the peritrophic matrix and the eggshell, suggesting molecular convergence among distinct extracellular matrices. More surprisingly, gene regulation, development, and proteolysis were statistically over-represented ontology terms for all non-cuticular matches in *Drosophila*. Searches against other arthropod genomes indicate that the motifs are taxonomically widespread.

**Conclusions:**

This survey suggests a more general definition for GYR and YLP motifs and reveals their contribution to several types of extracellular matrix. They may define sites of protein interaction with DNA or other proteins, based on ontology analysis. These results can help guide experimental studies on the biochemistry of cuticle assembly.

## Introduction

Like many extracellular matrix proteins, arthropod cuticular proteins frequently contain a large proportion of low-complexity sequence. Short repeats of characteristic amino-acid composition, such as glycine-rich and alanine/proline/valine-rich repeats, appear to have evolved independently in distantly related paralogs or different protein families [1], [2], [3]. Indeed, the presence of these repeats in a novel protein is sometimes used as evidence that it is cuticular in nature, although the sensitivity and specificity of these patterns for predicting gene ontology have not been evaluated. While the functional significance of these low-complexity sequences in cuticle is not well understood, hypotheses include modulating physical properties of the protein, serving as protein modification sites, or promoting specific protein-protein interactions. For example, short repeated motifs in the cuticular protein resilin contribute to protein elasticity [4]. Short repeated motifs of some chorion proteins are sufficient to induce self-assembly of amyloid fibrils [5], and the contribution of glycine and proline/hydroxyproline repeats to collagen aggregates has been extensively studied (reviewed by [6]). Post-translational modification sites are frequently marked by short conserved motifs in a variety of protein matrices (e.g. [7], [8]), and sites of cuticular protein modification or cross-linking are likely to be similarly conserved.

Given the abundance of arthropod genome data that has recently become available, there is both a need to better classify novel proteins with low-complexity sequence regions and an opportunity to identify functional elements in these regions. One bioinformatic approach to identifying candidate functional elements is to search for sequence blocks that are conserved among orthologous genes and relatively common among paralogous genes of a given family or ontological group. Motifs shared among nonhomologous genes may have arisen independently by convergent evolution or by domain shuffling, and suggest a biochemical feature of general importance.

A growing number of protein families have been identified that are structural constituents of cuticle, and their nomenclature has been reviewed recently by Willis (2010). In brief, cuticular protein names often include a “CP” prefix plus additional letters that denote domains or other sequence features (e.g., CPR for ‘cuticular protein with Rebers and Riddiford Consensus domain’ and CPLCG for ‘low-complexity cuticular protein with conserved glycines’). Other protein families follow different conventions, such as the Tweedle family, which is named for a mutant phenotype of the *TweedleD* gene in *Drosophila melanogaster* [9]. Family members of a given species often have species-level prefixes as well, e.g. AgamCPR1 and BmorCPR1 for (not necessarily orthologous) CPR proteins in *Anopheles gambiae* and *Bombyx mori*, respectively, but I will dispense with species designations in this paper.

The goal of this study was to systematically survey orthologous cuticular protein sequences of *Drosophila* for common, conserved sequence blocks. I focused on the CPR and Tweedle families because they are by far the largest cuticular protein families in *Drosophila,* they have a wide taxonomic distribution, and are defined by well-conserved domains that have demonstrated roles in cuticle assembly [9], [10], [11]. A distinction can therefore be made between the ‘consensus domain region’ and the ‘flanking regions’ for these gene families, which is not necessarily true of other cuticular protein families that are fewer in number and/or have lower levels of sequence conservation. The Tweedle family in particular is interesting because it represents an expansion in *Drosophila* (∼27 genes) of a small gene family of only two to four genes outside of the Diptera [9], [12]. The sequences flanking the conserved Tweedle domain show considerable variation in amino-acid composition that may contribute to their functional divergence.

## Methods

I used BioEdit [13] to identify conserved regions after masking the signal peptide and the family-defining domains of the CPR family (Pfam domain PF00379, slightly modified according to [14]), and the Tweedle family (Pfam domain PF03103). I used protein alignments from seven *Drosophila* species: *D. melanogaster*, *D. ananassae*, *D. pseudoobscura*, *D. willistoni*, *D. grimshawi*, *D. mojavensis*, and *D. virilis*; these annotations are detailed in [15]. I initially used relatively permissive criteria to define blocks: a minimum length of eight residues, with an average entropy of 0.4, at most two gaps of length one, and at most two sites with an entropy > 0.2. The entropy of an alignment site is a measure of variation in its state and a criterion by which sequence conservation can be objectively quantified: entropy is zero at invariant sites and has a maximum value of one when all possible states (residues) occur equally frequently (or proportional to the background composition). I generated an initial set of position-specific scoring matrices (PSSMs) from the resulting sequence alignments by using the BLOCKS server (http://bioinfo.weizmann.ac.il/blocks; [16]). I then used MAST (http://meme.sdsc.edu/meme4_3_0/cgi-bin/mast.cgi; [17]) to identify block PSSMs that were highly correlated with each other and thus largely redundant. Such blocks were combined if of equal length or else the shorter was eliminated. This process was repeated until no pair of blocks had a correlation coefficient greater than or equal to 0.6. I then used MAST to search *Drosophila* proteomes for stringent matches (P < 0.00001 for each motif match).

This initial search identified several partially overlapping motifs that were notably abundant. I therefore consolidated these matches into two motifs by hand-alignment of the overlapping regions and resubmitting these alignments to the BLOCKS server. I then repeated the search using a modified method so that other arthropod proteomes could be scanned in addition to *D. melanogaster*. These additional proteomes were: *Anopheles gambiae* [18], *Apis mellifera* [19], *Bombyx mori* [20], [21], *Daphnia pulex* (wfleabase.org), *Ixodes scapularis* and *Pediculus humanus* (www.vectorbase.org), and *Tribolium castaneum* [22]. For this extended search, I used the program ematrix [23] to identify an initial pool of peptides with at least one motif match at an expectation of 10^−4^. Additionally, at least one motif match per peptide was required to have the central tyrosine (see Results) given its biological relevance and invariance in the alignments that generated the motifs. This pool of candidates was then submitted to MAST with a statistical threshold of P < 0.00001. To approximate the number and strength of false positive matches, I also performed the motif search against *D. melanogaster* after randomizing each individual protein sequence, thereby maintaining the same sequence composition and length distribution.

In addition to collecting the number and architecture of motif occurrences from MAST, I used several bioinformatic strategies to annotate matching proteins. These included BlastP versus the *Drosophila melanogaster* proteome, Pfam domain (http://pfam.janelia.org; [24]) searches with Hmmer (http://hmmer.janelia.org), signal peptide prediction with SignalP [25], and gene ontology assignments with the Blast2GO program [26]. If several isoforms of the same locus were matched, only the first encountered was included in the data set. Additional annotation data were obtained from FlyBase (http://www.flybase.org).

## Results

### GYR- and YLP-like motifs are common in *Drosophila* cuticular proteins

**Figure 1:**
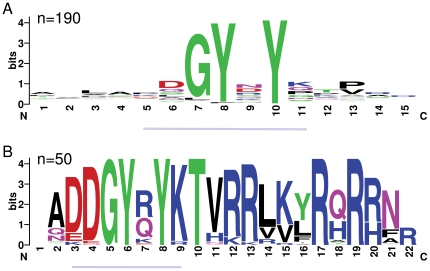
Comparison of sequence logos for Motif 1 and the GYR motif (PF02756). The number of sequences contributing to each sequence logo is indicated. A. Sequence logo of all *Drosophila* matches to Motif 1 (underlined portion) plus the four amino acids on either side. B. Sequence logo of all *Drosophila* matches to PF02756 from the Interpro web site (http://www.ebi.ac.uk/interpro/). The portion of the logo that corresponds to Motif 1 is underlined.

**Figure 2:**
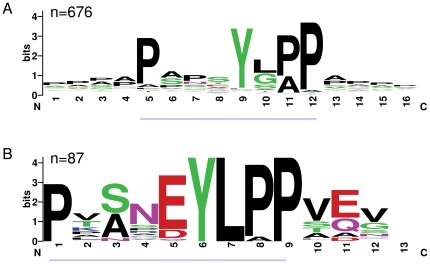
Comparison of sequence logos for Motif 2 and the YLP motif (PF02757). The number of sequences contributing to each sequence logo is indicated. A. Sequence logo of all *Drosophila* matches to Motif 2 (underlined portion) plus the four amino acids on either side. The sequence logo is based on slightly fewer (n = 676) than the total number of matches (n = 683, Table 2), because some matches lacked flanking sequence. B. Sequence logo of all *Drosophila* matches to PF02757 from the Interpro web site (http://www.ebi.ac.uk/interpro/). The portion of the logo that corresponds to Motif 2 is underlined. The underlined sequences differ in length because the latter is based on a gapped alignment.

Numerous motifs derived from conserved cuticular protein blocks had significant matches other than to the proteins they were derived from, yet two motifs were notably more common and transcended gene families (see below). I therefore focused on these motifs as strong candidates for having general roles in cuticular protein function, which are referred to hereafter as Motif 1 and Motif 2. While the initial sequence blocks identified were a minimum of eight residues, Motif 1 was reduced to seven residues in length because only these residues overlapped between several distinct but correlated blocks. Both motifs have a central, highly conserved tyrosine (**Figures 1** and **2**) and Motif 1 has another conserved tyrosine at the second position.

**Figure 3:**
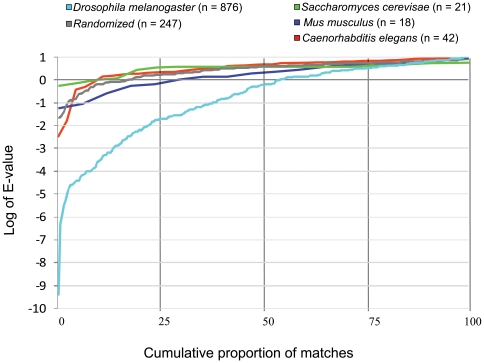
Distribution of motif E-values for matches to actual and randomized *D. melanogaster* sequence, and to non-arthropod model organisms. Each line represents MAST-calculated E-values of matches to the respective protein data set. The X-axis represents the cumulative percentage of matched proteins, whereas the total number of matches is indicated in the legend. In addition to having lower E-values, the total number of matches was substantially lower for the randomized and non-arthropod model organisms compared with *D. melanogaster*.

**Figure 3** shows the distribution of expectation estimates (E-values) for actual *D. melanogaster* matches compared with randomized data and with non-arthropod data. There were substantially fewer matches in the randomized data set: 87 versus 193 of Motif 1 and 160 vs. 683 of Motif 2. However, while a majority of *D. melanogaster* matches had E-values less than one, the possibility of false-positives appears nontrivial for E-values greater than ∼0.1 based on the E-value range of matches to the randomized data. Nonetheless, all matches with E-values ≤10 were retained for this analysis for two reasons: 1) to include lower scoring sequences that are homologous to more strongly matching proteins and align with them at motif sites, and 2) because there was no significant enrichment in gene ontologies in the randomized matches, unlike the actual data (see below). Curiously, there were more motif matches with E-values ≤10 in the randomized *D. melanogaster* sequence than in the non-arthropod model organisms *Saccharomyces cerevisae*, *Caenorhabditis elegans*, and *Mus musculus*, although the range of E-values was similar (**Fig. 3**).

Database searches revealed that Motif 1 is similar to the Pfam-annotated GYR motif (PF02756) and Motif 2 is similar to the Pfam-annotated YLP motif (PF02757). The names GYR and YLP refer to conserved amino acids in the Pfam models. However, both motifs described here are more general than the existing Pfam domains and match a greater number of proteins in *Drosophila*. **Figure 1A** shows a sequence logo representation of all *Drosophila* matches to Motif 1, as well as the four amino acids flanking the motif in either direction. **Figure 1B** shows a sequence logo representation of all *Drosophila* matches to PF02756 in the Interpro database. The overlapping positions of Motif 1 and PF02756 are indicated by grey lines in **Figure 1**. It can be seen in **Figure 1A** that the -1 position is comparatively conserved and could be included in an expanded version of Motif 1. **Figure 2** shows the equivalent comparison between Motif 2 and PF02757. These two formulations of the motif are of different lengths because the alignment underlying PF02757 includes a gap.

**Supporting Information File S1** summarizes the types of *Drosophila* proteins found to contain significant matches to Motif 1 and Motif 2. The majority of matched proteins had predicted signal peptides (130 of 190, or 68.4%). Proteins with multiple occurrences of Motif 2 were even more likely to have signal peptides (82 of 102 or 80.4%). CPR and Tweedle cuticular proteins were the most common protein families overall, as would be expected. Ten of 103 *CPR* genes and 23 of 27 *Tweedle* genes encoded proteins with one or more occurrences of either motif under the search parameters, as did all three members of the CPF cuticular protein family. One of the CPR proteins, CG1136, has lower overall similarity to other family members in *Drosophila* and was not named in previous surveys [15], [27], although it has a significant PF00379 domain. Almost all of these matches had at least one occurrence of Motif 2, whereas Motif 1 was less common. In fact, Motif 1 constituted only 19% of occurrences in all matched proteins. Two additional *Drosophila* proteins that match these motifs, Miniature and Dusky-like, are members of a small gene family involved in interactions between the apical membrane of the epidermis and the developing cuticle [28]. Taken together, the occurrence of these motifs in members of different protein families associated with cuticle suggests that they are functional sites involved in cuticular protein assembly.

### Tweedle and GCR1 proteins share a common motif architecture

**Figure 4:**
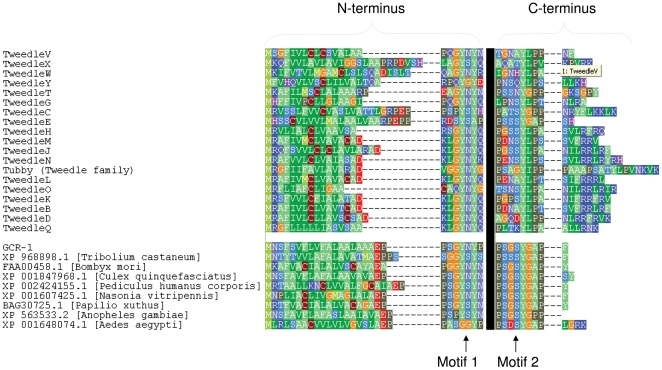
Alignment of the N-terminus and C-terminus of representative *Drosophila* Tweedle proteins with the *Drosophila* GCR1 protein and its inferred orthologues from several insect species. The N-terminal copy of Motif 1 and C-terminal copy of Motif 2 are indicated. The black line represents the intervening sequence between the two motifs that has been deleted for clarity.

Most Tweedle proteins exhibited a distinct motif pattern with an N-terminal occurrence of Motif 1 and one or more C-terminal occurrences of Motif 2 (**Figure 4**). This same pattern characterizes the glycine-rich protein GCR1 of *D. melanogaster* (CG2150) and its likely orthologs in other insect species (**Fig. 4**), which have no other recognized conserved domain. The GCR1 gene is highly expressed during *Drosophila* development [29], but its function is unknown. The motif structure shared with the Tweedle family suggests that GCR1 is cuticular, which is also consistent with the cuticle-like glycine repeats and signal peptide that are present. Embryonic *in situ* images in the Fly Express database [http://www.flyexpress.net/results.php?source=BDGP&search=CG2150&type=gene_images&gene=37631&page=1] further support this conclusion, showing strong expression of GCR1 in embryonic tissues that secrete cuticle, such as the head, esophagus, and pharynx.

### Motif 2 repeats are a common architecture exemplified by resilin

**Figure 5:**
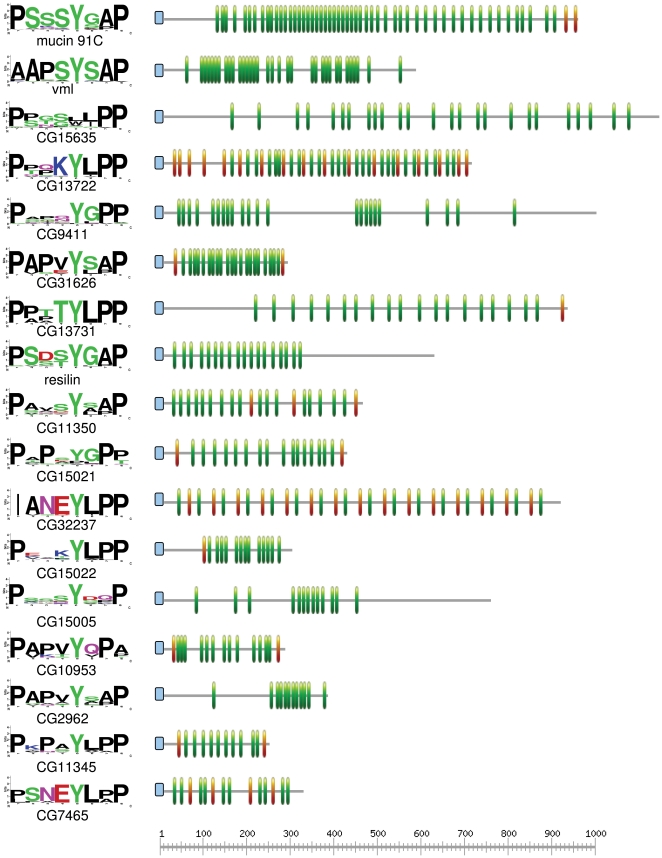
Motif architectures of *D. melanogaster* proteins with 10 or more copies of Motif 2. Motif 1 is indicated by orange symbols and Motif 2 is indicated by green symbols. Signal peptides are indicated by blue symbols. All proteins are diagrammed to the scale shown at bottom. The sequence logos at left represent the alignment of all copies of Motif 2 within that protein, illustrating the relative uniformity of motif copies within proteins versus among proteins.

Motif 2 typically occurred as regularly spaced repeats, frequently exceeding ten copies per protein (**Fig. 5** and **Supporting Information File S1**). One such protein is resilin, a highly elastic protein involved in the storage and release of energy during locomotion, such as jumping or flying [30]. Motif 2 in fact matches the resilin repeat that has been shown to confer elasticity [4], suggesting a general role of this motif in modulating the elasticity of structural proteins. This result is consistent with a recent analysis of resilin by Andersen [31] that identified two distinct repeat types, one of which overlaps with Motif2. Other proteins with many copies of Motif 2 are shown in **Figure 5** for comparison. A peritrophic-matrix mucin, mucin 91C, has the largest number of Motif 2 repeats identified in this study. Another annotated protein with a high number of repeats is the product of the vitelline-membrane-like gene (*vml*), a vitelline membrane protein that plays a role in dorsal-ventral patterning during embryogenesis [32]. The other proteins with ten or more copies of Motif 2 (**Figure 5** **)** all lacked functional annotations in FlyBase. Several of these proteins with ‘resilin-like’ repeats (i.e., Motif 2) were identified by Andersen as well [31].

Interestingly, the repeated copies of Motif 2 are relatively uniform in sequence within each protein but often differ among proteins, as indicated by the sequence logos in **Figure 5**. These compositional differences as well as variation in motif spacing might underlie biochemical or biomechanical specialization. Comparing the functions of biologically diverse proteins with similar motif architectures might be useful for the design of artificial peptides with specific biomechanical properties, similar to what has been accomplished with resilin [33]. However, a caveat to an adaptive interpretation is that protein repeats frequently evolve concertedly via unequal crossing over (e.g. [34], [35], [36]), resulting in repeat homogenization within proteins and divergence among proteins that can be due to drift alone. Distinguishing among adaptive and neutral hypotheses of repeat homogenization can be difficult and is beyond the scope of this study.

### Tandem arrays of low-complexity secreted proteins of unknown function

The chromosomal locations of matched *Drosophila* proteins reveal that many of the genes that encode them are physically clustered. Examples of three or more genes separated by less than 10 Kbp are marked in **Supporting Information File S1**. These include the majority of Tweedle genes, which were already recognized to occur in clusters [9]. Several other clusters consist of genes that encode very low complexity proteins, usually glycine or proline-rich, but which align poorly under default ClustalW parameters and are not recognizably homologous to each other (results not shown). The largest of these novel clusters includes ten genes on chromosome 3L. The remaining clusters contain three, four, and five genes, respectively. No Pfam domains were recognized in these clustered proteins, and no annotation terms were found in FlyBase. The sequence composition bias and presence of signal peptides suggest that these novel proteins may be secreted structural proteins of an undetermined type. It is also worth noting that only one gene, CG11349, encodes a mature protein with two cysteine residues, whereas the other predicted proteins contain cysteine only in the signal peptides. The absence of cysteines continues to be a hallmark of cuticular proteins [2]. Given the lack of evident homology, the evolutionary origins of such clusters are a puzzle that merits further study.

### Represented ontologies other than cuticle

A majority of motif matches were to proteins associated with ontological groups other than cuticle or that had no functional annotation. **Supporting Information File S2** contains output of the GOGraph Viewer of the Babelomics server (http://babelomics.bioinfo.cipf.es/; [26]), illustrating the distribution of ontologies (http://www.geneontology.org; [37]) that mapped to the set of matched Drosophila proteins. Graphs are shown for biological process and molecular function at several levels of resolution. These graphs collectively suggest an over-representation of particular classes of protein-protein interaction. These include terms related to structural biopolymers as expected, but also abundant were terms related to signal transduction, protein kinase activity, developmental processes, and proteolytic activity. **Supporting Information File S3** provides a post-hoc summary of gene ontologies that is based on edited biological-process and molecular-function terms downloaded from FlyBase as well as information from other sources, as described in **Supporting Information File S4**. This file condenses the more detailed annotations listed in **Supporting Information File S1**, and further illustrates the prevalence of regulatory genes, development genes, signal transduction genes, and non-cuticular extracellular matrix genes, in addition to structural genes of cuticle. GO term enrichment was tested statistically with the Fatigo tool [38] after removing known cuticular protein genes from the test sample. **Table 1** shows the terms that were significantly over-represented in the sample relative to all *D. melanogaster* genes. The results were consistent with the patterns described above, in that terms related to gene regulation (“DNA binding”), development, and proteolytic (“carboxypeptidase”) activity were significantly enriched. Thus, while many matched proteins lacked functional characterization, the annotations that are extant suggest a general role for GYR- and YLP-like motifs in several processes with potential biochemical similarities.

**Table 1:** Statistically over-represented GO terms for the set of *Drosophila* genes encoding proteins that match Motif 1 and Motif 2, after excluding known cuticular proteins.

Database level	Database term	Sample frequency	Frequency in all *Drosophila* proteins	P value	Adjusted P value
GO molecular function at level 4	DNA binding (GO:0003677)	74.13%	25.87%	0.0002	0.028
GO molecular function at level 8	carboxypeptidase A activity (GO:0004182)	95.61%	4.39%	0.0002	0.022
GO biological process at level 3	multicellular organismal development (GO:0007275)	67.12%	32.88%	0.0013	0.038
	cell proliferation (GO:0008283)	88.06%	11.94%	0.0024	0.038
	sexual reproduction (GO:0019953)	75.76%	24.24%	0.0021	0.038
	anatomical structure development (GO:0048856)	69.59%	30.41%	0.0013	0.038
GO molecular function at level 4	DNA binding (GO:0003677)	74.13%	25.87%	0.0002	0.028

Output is from the Fatigo web tool [38].

### Frequency of occurrence in other arthropod species

**Table 2** shows the number of genes of seven arthropod species that encode proteins that match Motif 1 or Motif 2 at the same statistical threshold used for *D. melanogaster* (P < 1.0E-5 for individual occurrences). The number of matched proteins varied substantially among species, but did not follow a clear phylogenetic pattern. The beetle *T. castaneum* had the smallest number of matched genes (23), but the highest number of Motif 2 occurrences per match (5.1) on average. The waterflea, *Da. pulex*, had a large number of matches, but the gene complement of that species is roughly twice that of most insects [39]. Matches in *A. gambiae* included the CPLCG family of cuticular proteins [12], which has homologues in *Drosophila* that were not detected by the motif search. Twenty-one *Da. pulex* matches had collagen domains, whereas only two collagen domain proteins were found in the other species searched. While it is of interest to know how many of the proteins matched in different species are orthologous, a systematic determination of orthology across distantly related species can be challenging and is beyond the scope of this study.

**Table 2:** Number of motif matches in eight arthropod species.

Species	Matched genes	Total Motif 1	Total Motif 2
*Anopheles gambiae*	116	77	276
*Bombyx mori*	160	88	208
*Daphnia pulex*	228	68	646
*Ixodes scapularis*	69	22	98
*Pediculus humanus*	81	42	189
*Apis mellifera*	70	51	159
*Tribolium castaneum*	23	25	118
*Drosophila melanogaster*	190	193	683

**Figure 6:**
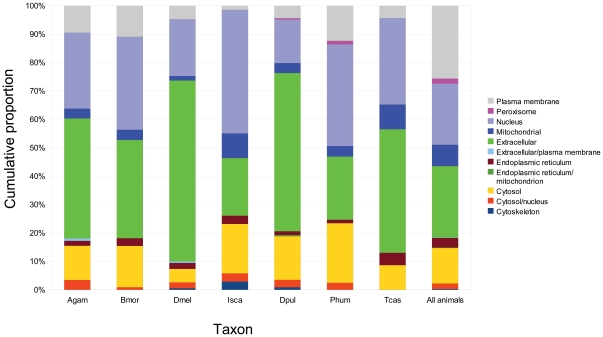
Predicted localization of motif-matching proteins by taxon. Columns represent the proportion of matched proteins that are predicted by WoLF-PSORT [40] to localize to the various cellular and extracellular regions indicated by the legend. For all arthropod species, the combined proportion of extracellular and nuclear-localizing proteins is higher than in animals generally [41], but the fraction that is predicted to be extracellular is highest in *Drosophila melanogaster* and *Daphnia pulex*. Species codes are as follows: Agam  =  *Anopheles gambiae*, Bmor  =  *Bombyx mori*, Dmel  =  *D. melanogaster*, Isca  =  *Ixodes scapularis*, Dpul  =  *Da. pulex*, Phum  =  *Pediculus humanus*, and Tcas  =  *Tribolium castaneum*.

Signal peptides were substantially less abundant among matches in other arthropods overall (280/698 or 40%), which may reflect biological differences among species, more false-positive matches outside of *Drosophila*, and/or variation in annotation quality. I therefore used WoLF-PSORT [40] to estimate the proportion of matched proteins that can be localized to various cellular compartments or that are extracellular. This provides an additional measure of the extent to which matches are enriched in extracellular matrix proteins, and the consistency of cellular localization across diverse species. **Figure 6** shows that for all species the proportion of matched proteins that are either nuclear or extracellular is greater than for animals generally [41]. However, the proportion that is extracellular is variable and clearly highest in *D. melanogaster*. Together, these results indicate that the abundance of extracellular matrix proteins among matches is lower in other arthropods than in *D. melanogaster*.

## Discussion

This study identified two motifs from conserved blocks of orthologous sequence that occur in multiple cuticular protein families of *D. melanogaster*. Motif 1 and Motif 2 overlap with and expand upon the existing YLP and GYR motifs in Interpro. These motifs are likely to be functionally important for cuticle formation because they 1) derive from conserved orthologous blocks, 2) occur in different protein families known to be involved in cuticle (e.g., Tweedles, CPRs, CPFs, *A. gambiae* CPLCGs) or cuticle-associated processes (Dusky/Miniature), and 3) have conserved tyrosines. Several potential roles of the conserved tyrosines in the functions of these motifs can be considered. For example, tyrosines are likely to contribute to chitin binding by the R&R Consensus domain of CPR proteins [42], and so may also interact with chitin outside of that domain. A possible role in protein cross-linking is suggested by the observation that tyrosine bridges underlie the cross-linking of resilin [43]. The conserved tyrosines could also be phophorylation targets of tyrosine kinases, as noted by the Interpro annotations of the GYR and YLP motifs. This possibility is further suggested by the fact that the Interpro YLP annotation includes the human protein ErbB4, a receptor tyrosine protein kinase. Other modifications such as glycosylation are possible, although tyrosine glycosylation is believed to be rare [44]. Alternatively, these motifs may not be covalently modified but instead contribute to protein folding. However, the roles of these motifs remain to be ascertained directly by biochemical and functional analysis, and it seems premature to assume that all motif copies have the same function. For example, Andersen notes that while resilin and some mucins contain similar repeats (i.e., Motif 2), the biophysical properties of the mature proteins are very different due in part to dityrosine cross-linking of the former and glycosylation of the later [31].

Although derived from the CPR and Tweedle cuticular protein families, the majority of *Drosophila* matches were to non-cuticular or unannotated proteins. The ontologies of these additional matches suggest that GYR- and YLP-like motifs are of general importance in certain types of protein interaction, particularly during development. Annotated proteins include components of extracellular matrix assembly and morphological patterning, signal transduction, and gene regulation. Some of these categories are intuitive candidates for biochemical convergence with cuticular proteins, such as the nonhomologous chitin-binding proteins of the peritrophic matrix. Two peritrophic matrix mucins were identified in the search (mucin related 2B [CG14796] and mucin 91C [CG7709]), along with two proteins with peritrophic chitin-binding domains (**Supporting Information File S1**). Interestingly, mucins as a group appear to have roles in *Drosophila* development as well [45]. Another matrix that has frequently been noted to share structural and biochemical similarities to cuticle is the chorion and vitelline membrane, which together comprise the eggshell. Both cuticle and eggshell are comprised of helicoidal lamellae that self-assemble through a liquid-crystal phase (e.g., [46], [47], [48]). It is therefore intriguing that four eggshell proteins contained motif matches.

An important caveat to the analysis of short sequence motifs is that the trade-off between sensitivity and specificity is likely to be particularly acute, given their inherently low information content. The partial overlap in E-values between matches to actual versus randomized *D. melanogaster* protein sequences (**Fig. 3**) clearly indicates that false-positives are of concern, and there is little basis to judge the rate of false-negatives. However, the total number of motif occurrences in the randomized data set was much lower (247 vs. 876, or 28.2%). Furthermore, the recurrence of motif architectures among homologous and non-homologous proteins, such as the Tweedle/GCR1 and resilin-like patterns, provides independent support for the biological significance of these motifs, as does the high frequency of signal peptides. Analysis of the secondary-structure context in which these motifs are embedded might provide additional biological information that could increase the specificity/sensitivity of motif detection. Looking forward, a test of the conclusions of this analysis is implicit in the large number of matched *Drosophila* proteins that remain to be annotated, as well as in the rapidly expanding sequence space of arthropod proteomes. Ultimately, reverse-genetic and biochemical studies will be needed to confirm whether motif matches are functionally related, as is true of any motif analysis. For example, proteomic studies of *Drosophila* cuticle analogous to the work that has been done in *Anopheles* [49] would be a relatively rapid way to confirm whether some matched proteins of unknown function are in fact cuticular components.

The identification of candidate functional elements shared among homologous and non-homologous genes bears on the topic of how multigene families diversify. Insect cuticular proteins are often members of large multigene families, with numerous expansions specific to particular lineages (e.g. the CPR, Tweedle, and CPLCG families). Identifying functional elements outside the core conserved sequence of a multigene family can suggest processes by which paralogous genes are retained. The level of sequence divergence amongst orthologous CPRs, for example, is often low outside of the conserved chitin-binding domain despite a high level of divergence among paralogues, indicating that these regions are functionally constrained. The demonstration that resilin repeats, which are a strong match to Motif 2, are sufficient to generate elasticity in artificial peptides [4] further highlights the contribution of short sequences to functional novelty. Such short dispersed elements could reasonably overshadow the conserved domain in contribution to the fitness of a particular gene. Under this scenario, dispersed functional elements could reduce constraint on an existing complex domain, fostering the acquisition of new functions for that domain. In this way, even evolutionarily labile motifs that may be present in only a few members of a multigene family could act as a bridge to the evolution of new variants of the complex domain. Alternatively, the complex domain could be lost entirely, resulting in a novel low-complexity gene lineage. This latter scenario is supported by the fact that the *D. melanogaster resilin* produces an alternative transcript (isoform B) with a severely truncated R&R Consensus. Whether such hypotheses can explain evolutionary patterns within insect development is becoming increasingly accessible to analysis, given the emerging phylogenetic richness of genomic data in taxa such as *Drosophila* and mosquitoes.

## Supporting Information

File S1 Spreadsheet of all matched *Drosophila* proteins and associated annotation data, including Pfam annotations, motif architectures from MAST, and signal peptide predictions.
(0.37 MB XLS)

File S2 GOGraph Viewer diagrams of ontology terms.
(0.70 MB PPT)

File S3 Table summarizing the ontologies of *Drosophila* matches to Motif 1 and Motif 2, based on ontology terms, Pfam domains, and the literature. Terms drawn from Supporting Information File S4.
(0.06 MB DOC)

File S4 Edited ontology terms used in Supporting Information File S3, listed by gene.
(0.13 MB XLS)
