# Supplementary material for: The Distribution of GYR- and YLP-Like Motifs in Drosophila Suggests a General Role in Cuticle Assembly and Other Protein-Protein Interactions
Source: PLoS One. 2010 Sep 2;5(9):e12536. doi: 10.1371/journal.pone.0012536 (PMC2932725; doi:10.1371/journal.pone.0012536)
Supplement: File S2 — GOGraph Viewer diagrams of ontology terms. (0.70 MB PPT) [file pone.0012536.s002.ppt]

## Slide 1
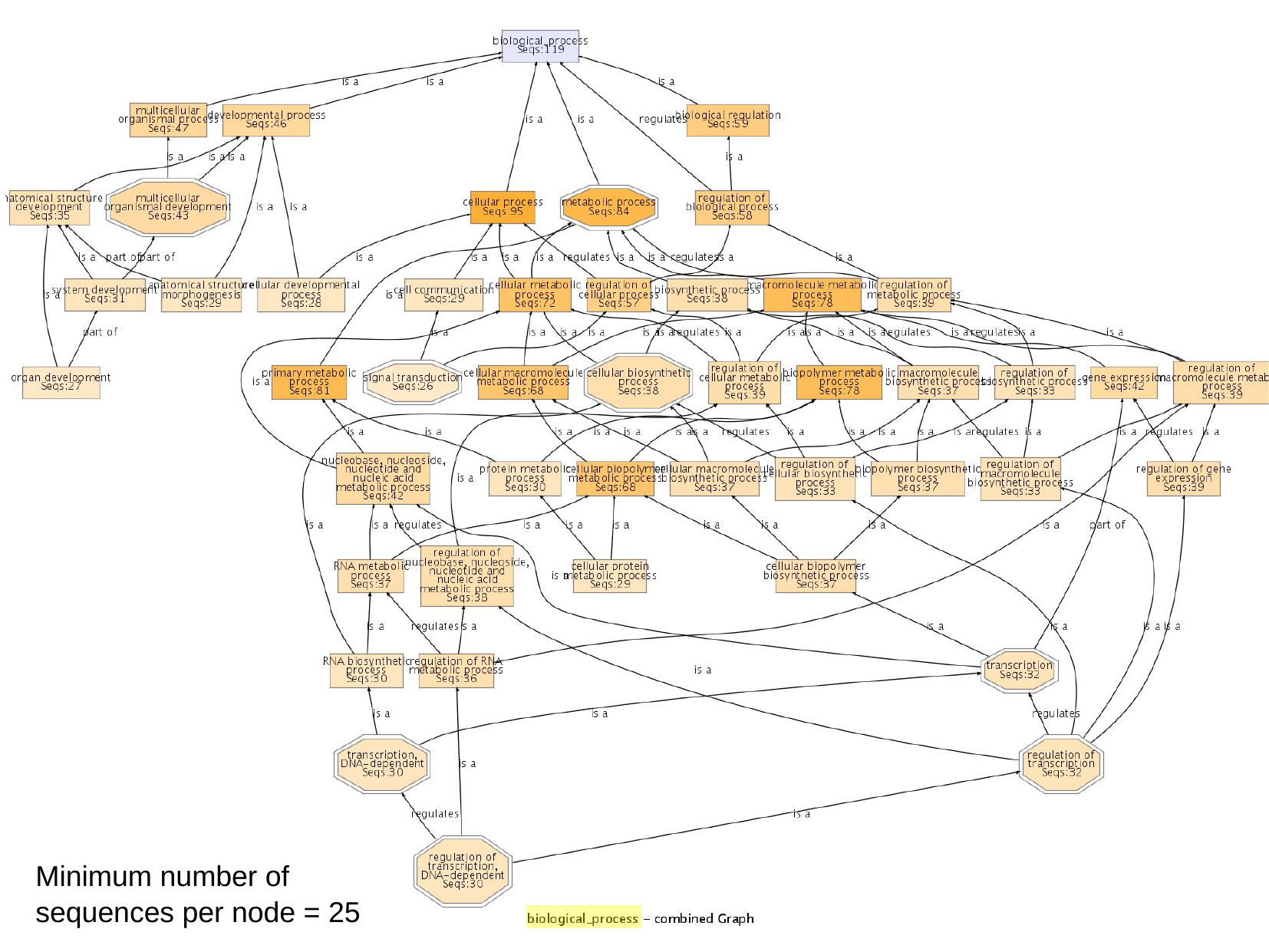

Minimum number of sequences per node = 25

## Slide 2
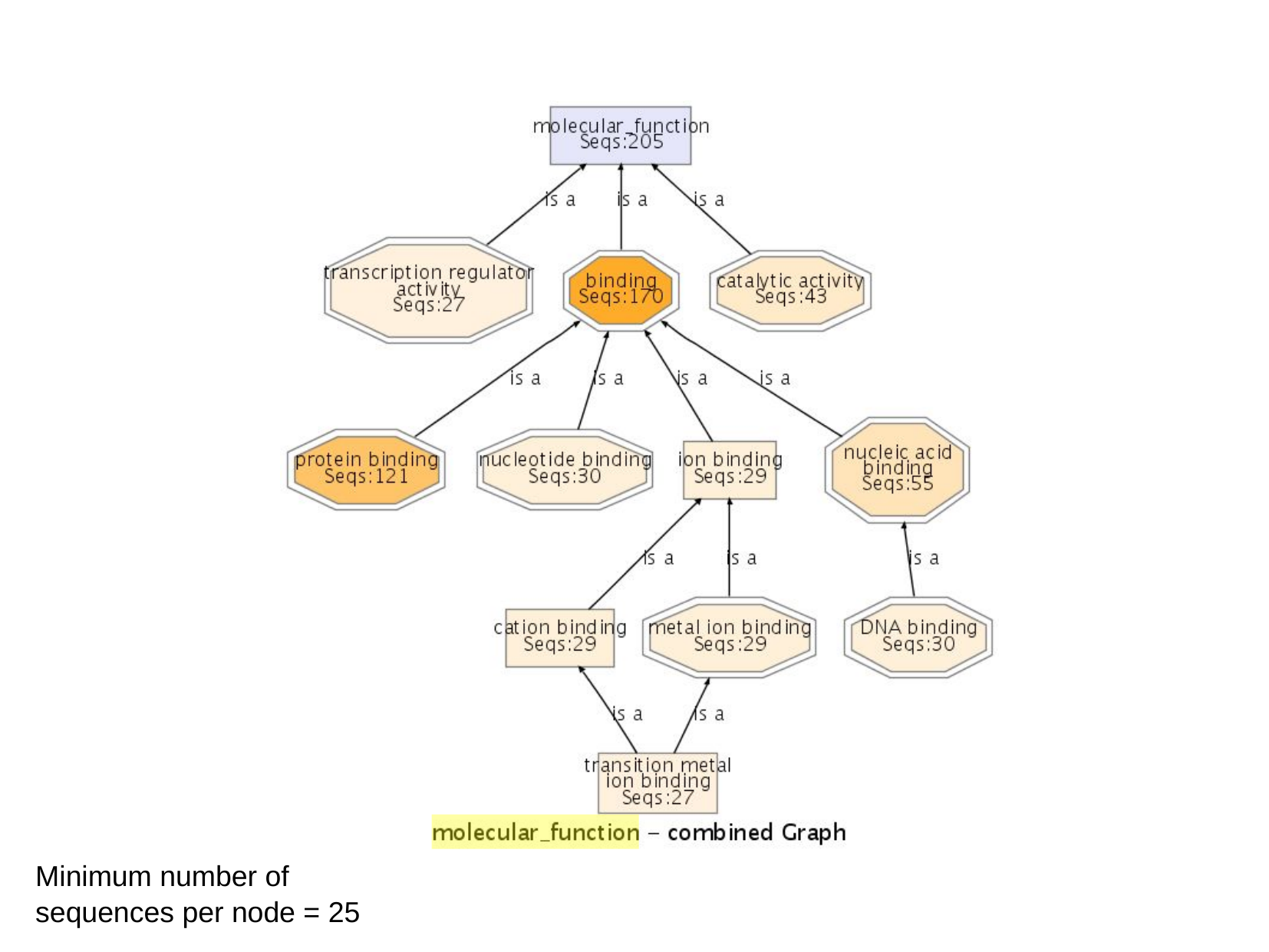

Minimum number of sequences per node = 25

## Slide 3
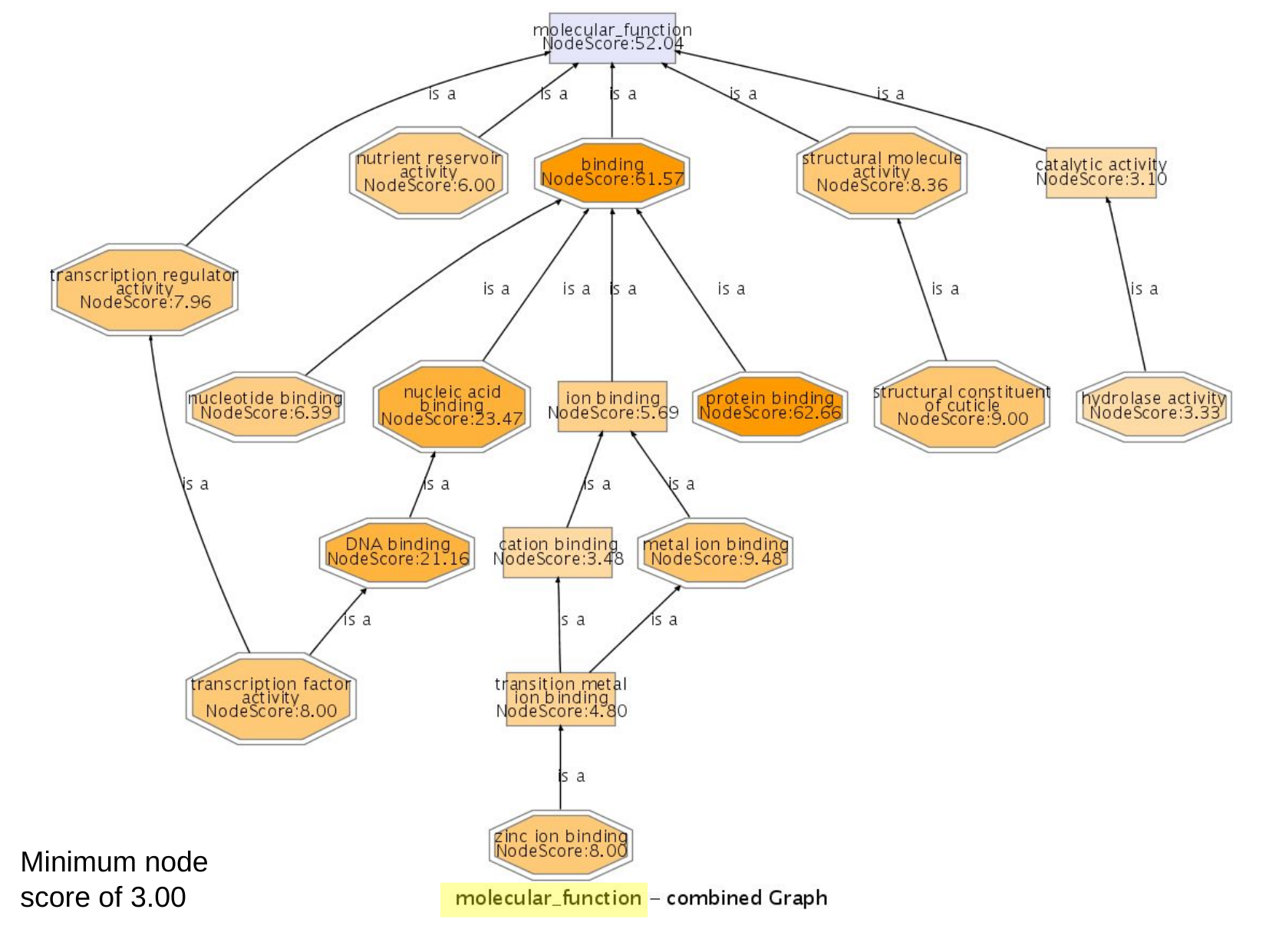

Minimum node score of 3.00
