# Supplementary material for: The Distribution of GYR- and YLP-Like Motifs in Drosophila Suggests a General Role in Cuticle Assembly and Other Protein-Protein Interactions
Source: PLoS One. 2010 Sep 2;5(9):e12536. doi: 10.1371/journal.pone.0012536 (PMC2932725; doi:10.1371/journal.pone.0012536)
Supplement: File S3 — Table summarizing the ontologies of Drosophila matches to Motif 1 and Motif 2, based on ontology terms, Pfam domains, and the literature. Terms drawn from Supporting Information File S4. (0.06 MB DOC) [file pone.0012536.s003.doc]

Summary of *Drosophila* matches to Motif 1 and Motif 2, based on ontology terms, Pfam domains, and the literature. Terms drawn from Supporting Information File S4. APV = alanine, proline, and valine.

| Category | Description | Number of genes |
| --- | --- | --- |
| Matrix protein | structural constituent of cuticle | 39 |
|  | low complexity APV-rich with signal peptide | 9 |
|  | structural constituent of eggshell | 4 |
|  | peritrophic matrix | 4 |
|  | low complexity glycine-rich with signal peptide | 2 |
|  | extensin-like domain | 1 |
|  | structural constituent of cytoskeleton | 1 |
| Development | dorsal/ventral axis specification | 3 |
|  | wing morphogenesis | 2 |
|  | anterior/posterior pattern formation | 1 |
|  | axon guidance | 1 |
|  | central nervous system development | 1 |
|  | compound eye photoreceptor development | 1 |
|  | dendrite morphogenesis | 1 |
|  | determination of adult lifespan | 1 |
|  | embryonic development via the syncytial blastoderm | 1 |
|  | epidermis development | 1 |
|  | female germ-line sex determination | 1 |
|  | germ cell migration | 1 |
|  | imaginal disc-derived leg morphogenesis | 1 |
|  | imaginal disc-derived wing vein specification | 1 |
|  | long-term memory | 1 |
|  | mesoderm development | 1 |
|  | regulation of cell shape | 1 |
|  | spatzle neurotrophin | 1 |
|  | muscle thick filament assembly | 1 |
| Regulatory | regulation of transcription | 3 |
|  | chromatin remodeling | 1 |
|  | DNA binding | 1 |
|  | methylation | 1 |
|  | negative regulation of mRNA translation | 1 |
|  | negative regulation of transforming growth factor beta receptor signaling pathway | 1 |
|  | neurotransmitter secretion | 1 |
|  | regulation of alternative nuclear mRNA splicing, via spliceosome | 1 |
|  | translational initiation | 1 |
| Signal transduction | epidermal growth factor receptor signaling pathway | 1 |
|  | insulin receptor signaling pathway | 1 |
|  | taste receptor activity | 1 |
|  | signal transducer activity | 1 |
|  | small GTPase mediated signal transduction | 1 |
|  | transmembrane receptor | 1 |
| Other | proteolysis | 8 |
|  | transmembrane transport | 1 |
|  | zinc ion binding | 3 |
|  | lipid transport | 1 |
|  | phospholipid metabolic process | 1 |
|  | protein amino acid phosphorylation | 1 |
|  | phosphopantetheine binding | 1 |
